# Visual Field Test With Gaze Check Tasks: Application in a Homonymous Hemianopic Patient Unaware of the Visual Defects

**DOI:** 10.3389/fneur.2021.682761

**Published:** 2021-06-02

**Authors:** Katsuei Shibuki, Tsuyoshi Yokota, Akane Hirasawa, Daisuke Tamura, Shin Hasegawa, Takashi Nakajima

**Affiliations:** ^1^Department of Clinical Laboratory, Kashiwazaki General Hospital and Medical Center, Kashiwazaki, Japan; ^2^Brain Research Institute, Niigata University, Asahi-Machi, Chuo-Ku, Niigata, Japan; ^3^Department of Rehabilitation, Kashiwazaki General Hospital and Medical Center, Kashiwazaki, Japan; ^4^Department of Internal Medicine, Kashiwazaki General Hospital and Medical Center,Kashiwazaki, Japan; ^5^Department of Neurology, National Hospital Organization Niigata National Hospital, Kashiwazaki, Japan

**Keywords:** visual field test, visual cortex, homonymous hemianopia, eye movement, gaze control

## Abstract

Gaze control is required for applying visual stimuli to a particular area of the visual field. We developed a visual field test with gaze check tasks to investigate hemianopia. In this test, participants must report the presence or absence of visual stimuli when a small object at the fixation point vibrates. Trials in the absence of visual stimuli were used as gaze check tasks, since the vibration could be observed only when the gaze was directed at the fixation point. We evaluated the efficacy of our test in four control participants and one patient with homonymous hemianopia who was unaware of the defects in the left visual field. This patient presented hemianopia in the test with gaze check tasks, but not when the gaze check tasks were omitted. The patient showed spontaneous gaze movements from the fixation point to the upper left direction, as well as scanning of the left visual field during the test without gaze check tasks. Thus, we concluded that the visual defects in this patient were compensated in daily life by spontaneous eye movements coordinated with visual information processing. The present results show the usefulness of the visual field test with gaze check tasks.

## Introduction

Visual information is received by the cerebral cortex via the lateral geniculate body and primary visual cortex, and this pathway enables us to see the external world (1, 2). If the hemilateral occipital lobe, including the visual cortex, is damaged, the cerebral cortex cannot receive visual information from the contralateral visual field, causing homonymous hemianopia (3–5). Cortical visual impairment after cerebral infarction ameliorates spontaneously in patients who undergo quantitative perimetry as part of their routine care (6). It has also been reported that recovery may be facilitated by early rehabilitation in small uncontrolled and/or unmasked studies (7, 8). Transient dysfunction in the cortical areas surrounding the infarct may recover spontaneously (6) or with compensatory neuronal plasticity in the remaining cortical areas (9–11). Recovery can be observed even in patients who have had a cortical infarction over 5 years ago (12). The recovery process should be monitored by a visual field test in which various visual stimuli are applied to a particular part of the visual field. However, it is difficult for conventional perimetry to apply various visual stimuli and analyze the higher visual functions that strongly depend on stimulus parameters (13–15). Therefore, we developed a computer-based visual field test that can test visual functions by presenting various stimuli repeatedly, including moving grating patterns and letters, at specific parts of the visual field. To test a specific part of the visual field, the patient has to look at the fixation point. Therefore, the reliability of the results is confirmed only after checking the gaze control quality. Although it is possible for the examiner to visually confirm the gaze of the patient, it is laborious to repeatedly perform visual inspection during rehabilitation. In this study, we combined visual field tests with gaze check tasks that can be answered only when the patient looks at a small object at the fixation point. If the performance in the gaze check tasks was insufficient, the visual field test results were discarded. In practice, such conditions work as a pressure for the patient to concentrate on gaze control, and the results are discarded only when the patient is exhausted.

When the occipital lobes, including the visual cortex, are impaired, the patient could be unaware of the visual defect in rare cases (16–19). The mechanism of this condition, known as Anton syndrome, is not well-known, and it is more likely to occur when bilateral occipital lobes are impaired (18, 19). In homonymous hemianopia, the visual field opposite to the hemilateral cortical damage is impaired, but the visual defect is sometimes compensated by eye movements (20–22). We investigated a patient with homonymous hemianopia with left visual field defects using our visual field test. This patient presented hemianopia in the Goldmann perimetry but was unaware of the visual defects. The patient read approximately 150 books and watched 30 movies in theaters in a year. The apparent discrepancy between the hemianopia and the high level of vision sufficient for daily life could be explained by two possibilities. The apparently good visual functions could be due to anosognosia or intracerebral information processing mechanisms such as filling-in (23). Alternatively, the visual field defect could be compensated by eye movements (20–22). The results obtained using the present visual field test supported the latter possibility. For this patient to be unaware of the visual field defects, intimate coordination between eye movements and visual information processing would be required. Acquiring such coordination can be an effective therapeutic strategy to restore visual functions sufficient for hemianopic patients to be relieved of the visual defects in daily life.

## Methods

### Participants

The four control participants comprised of one woman (27 years old) and three men (67, 56, and 23 years old). The 67-year-old man wore reading glasses and the 23-year-old man wore near glasses during the visual field test. The data obtained from the four control participants were averaged. Similarly, the data of eight visual fields in the four control participants were averaged in some experiments. When one control was selected for comparison, the data obtained from the 67-year-old man were used. The homonymous hemianopic patient in this study was a 66-year-old man who had suffered from a cerebral infarction in the right occipital lobe, including the visual cortex, 12 years back. He visited Kashiwazaki General Hospital and Medical Center every 2 months and received cilostazol treatment to prevent further cerebral infarction. This patient wore near glasses during the visual field test.

### Visual Field Test

**Figure 1:**
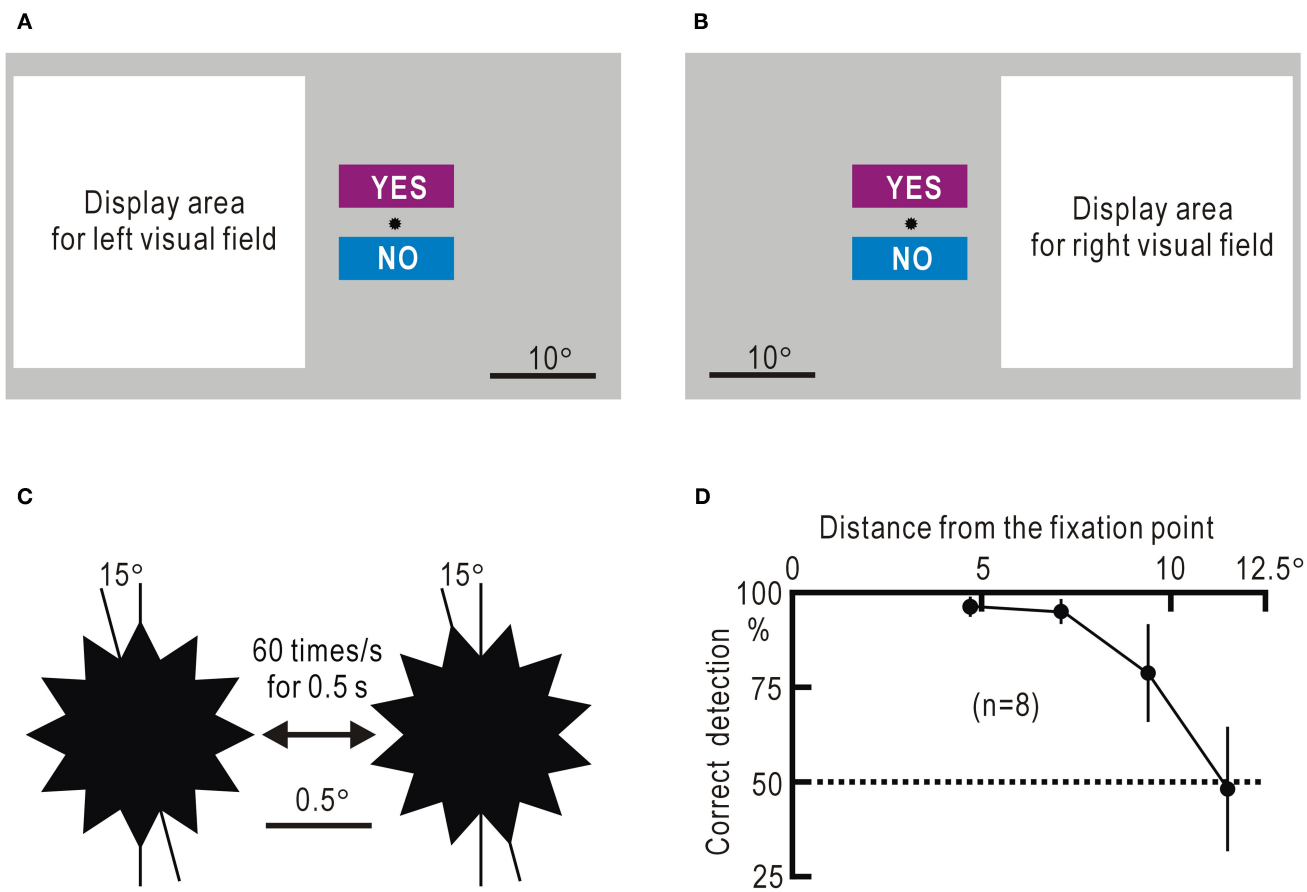
**(A)** Screen image used to test the left visual field. The white square represents the display area in which various stimuli are presented. The small star is placed at the fixation point. The rectangles labeled “YES” and “NO” are areas to which the participant responds by touching or clicking with a mouse. **(B)** Screen image used to test the right visual field. **(C)** Enlarged view of the dodecagonal star at the fixation point. The star rotationally vibrates for 0.5 s at a speed of 60 times/s with an angle of 15°. **(D)** Percentage of correct detection of the remote star vibration plotted against the distance between the object and the fixation point. The mean ± SEM of the eight visual fields in four control participants is shown.

We programmed the visual field test using Visual Basic 2019 (Microsoft). The visual field test was performed using a small computer/tablet (Surface Pro 7, Microsoft), considering bedside use. However, in this study, the video output from the computer was displayed on a 23.8-inch LCD touch monitor (P 2418 HT, Dell), so that participants sitting in front of the monitor at a distance of ~50 cm could respond to the test by touching the particular sections designed on the monitor or clicking on them with a mouse. Homonymous hemianopia often shows macular sparing (24). Therefore, we presented visual stimuli in a square display area, 8.5° away from the fixation point, where a small star was placed as the gaze target (Figures 1A,B). The visual stimulus was presented as a still image appearing for 0.5 s or as a movie for 0.5 s at 60 frames/s in a 27.5° square display area (Supplementary Video 1). The fixation point was marked with a dodecagonal star with a diameter of 1.1°. The star rotationally vibrated at 60 Hz with an amplitude of 15° for 0.5 s (Figure 1C), and visual stimuli were directed at the display area during the 0.5 s. In each session, 10 trials with visual stimuli and five without visual stimuli were conducted in random order for 15 trials. The trials without stimulus presentation, which were randomly intermingled in trials with stimulus presentation, served as gaze check tasks. Even if the visual field function corresponding to the display area is impaired, the gaze check tasks can be correctly answered when the gaze is directed at the fixation point. The participants touched or clicked the rectangle labeled “YES” when they sensed the stimulus and “NO” when they did not, during the star vibration at the fixation point (Figures 1A,B, Supplementary Video 1). A touch or click within 2 s after the onset of the star vibration was considered as a response, while other touches and clicks were ignored. Furthermore, only the first touch or click within the 2-s period was accepted as a response. The intervals between trials were randomly determined between 4 and 6 s. The results of a session were discarded, unless four or five “NO” responses were obtained in the five trials without stimulus presentation. Failure to obtain “NO” responses was sometimes observed when the participants were exhausted and overlooked the star vibration at the fixation point. The visual field test was interrupted when the participant was judged to be exhausted.

### Visual Stimuli

We used six types of visual stimuli (Supplementary Video 1): slow stimulation, a disk inscribed in the display area of 27.5° square gradually appeared over 0.5 s; fast stimulation, a disk inscribed in the display area suddenly appeared and gradually disappeared over 0.5 s; looming stimulation, a disk inscribed at the midpoint of the side far from the fixation point of the display area increased in diameter by 5% for each frame, maintaining the inscribed state until the disk inscribed four sides of the display area after 0.5 s; static stimulation, five disks with a diameter of 9.2° and arranged pentagonally appeared for 0.5 s; grating stimulation, vertical stripes of square waves at 5.5°/cycle wide appeared for 0.5 s and moved right or left at a speed of 2.0 cycles/s (11°/s); and letter stimulation, one of 46 Japanese hiragana letters in font size 620 points appeared for 0.5 s. When the grating stimulation was presented, an option for judging the moving direction could be added. The moving speed was adjustable in the range of 0.03–10 cycles/s. When the letter stimulation was presented, an option to select the presented letter from five different letters was added. The font size of the letter initially displayed in the display area was adjustable in the range of 18.6–620 points.

### Analysis of Eye Movements and Gaze Shifts

Eye movements of the hemianopic patient and a control participant during the visual field test without gaze check tasks (Supplementary Videos 2, 3) were recorded using a camera (D7000, Nikon). The gaze shifts were measured using an eye tracker (Tobii Pro Nano, Tobii Technology, Stockholm, Sweden) and software (Tobii Pro Lab), and the results were superimposed on the screen images in the visual field test.

### Statistics

The averaged data were presented as mean ± SEM. For the statistical test in some experiments, the χ^2^ test was performed using Excel (Microsoft). The *P*-values in multiple comparisons were corrected using the Bonferroni method.

## Results

### Gaze Control During the Visual Field Test

To estimate the extent of gaze control during the visual field test, we measured the probability of detecting the star vibration that was placed away from the fixation point in four control participants (eight visual fields on either side). In this experiment, a session had 10 trials with remote star vibration and randomly mixed five trials without remote star vibration. Control participants were asked to respond with “YES” when they felt remote star vibration and “NO” when they did not. The probability (P) of the correct responses of “YES” was estimated as:

$$\begin{array}{r} 100\%-(\text{false}-\text{negative probability}+\\ \text{false}-\text{positive probability}). \end{array}$$

The false-negative probability was estimated from the percentage of trials with remote star vibration that were mistakenly responded with “NO.” The false-positive probability was estimated from the percentage of trials without remote star vibration that were mistakenly responded with “YES.” The estimated *P*-value was 96.3 ± 2.6% at 4.7°, and it tended to decrease as the distance increased (Figure 1D, Supplementary Table 1). In this argument, it was assumed that participants were properly looking at the fixation point during the experiment. In fact, the four control participants were tested in 480 trials in this experiment, and they responded with “YES” or “NO” in all trials.

The visual field test was applied in the four control participants. One session of our visual field test was composed of 15 trials (10 trials with stimulus presentation and 5 trials without stimulus presentation). If the correct answer “NO” was not obtained in five or four of the five trials without stimulus presentation, the results of the other 10 trials with stimulus presentation were assessed as unreliable and were discarded. Of the 152 sessions performed by the four control participants with fulfilled criteria, 144 sessions included five correct answers of gaze check tasks in each session, and the results of the other eight sessions included four correct answers in each session. When the probability of detecting the star vibration at the fixation point is P, the probability of responding with “NO” in all the five gaze check tasks is P^5^. The probability of responding with “NO” in four of the five gaze check tasks was 5 × (1–P) × P^4^. Thus, the ratio of these two probabilities, P/[5 × (1–P)], can be measured as the ratio of the number of sessions with five “NO” responses to the number of sessions with four “NO” responses, and the *P*-value can be calculated. The estimated *P*-value was 98.9%. Since this value is >96.3%, we concluded that the gaze was fixed within a range of ~4.7° from the fixation point during the visual field test. The gaze check task also served to assess the contamination by false-positive responses. Of the 760 trials in the gaze check tasks, there was no false-positive trial with “YES” response. Therefore, we assumed that the contamination by false-positive responses was negligible.

**Figure 2:**
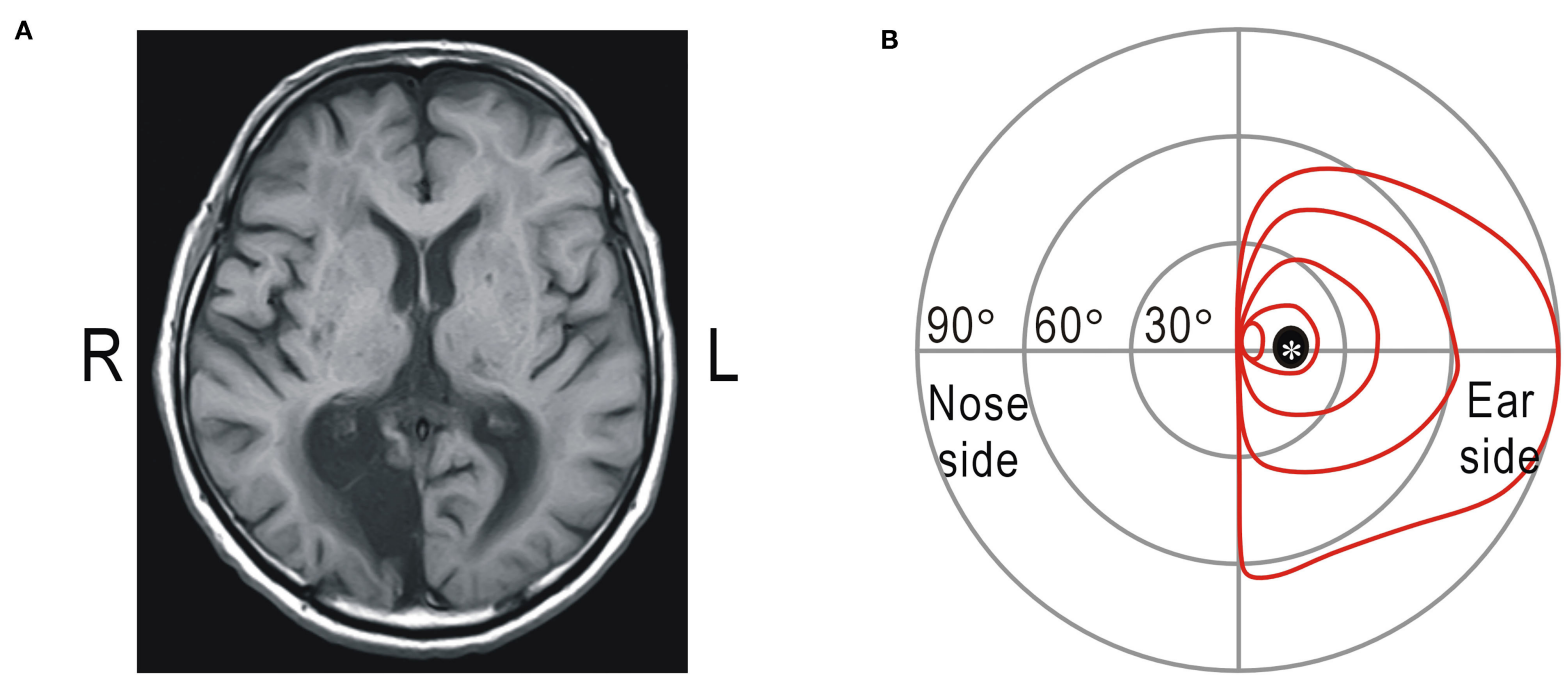
**(A)** Magnetic resonance imaging of the brain. Cerebral infarction noted in the right occipital lobe including the visual cortex (R: right; L: left). **(B)** Results of Goldmann perimetry of the right eye. *Mariotte blind spot.

The visual field test was also applied in a homonymous hemianopic patient. The patient was a 66-year-old man whose right occipital lobe, including the primary visual cortex, had been impaired by infarction 12 years ago (Figure 2A). Goldmann perimetry revealed that the left visual field contralateral to the cortical damage was unresponsive to visual stimuli (Figure 2B). There was no macular sparing due to extensive cortical damage. The patient maintained a high level of visual function and was unaware of the visual defects in daily life; he read approximately 150 books and watched 30 movies in theaters in a year. Of the 24 sessions performed by the patient with the fulfilled criteria, the results of 23 sessions included five correct answers of gaze check tasks in each session and that of the remaining one included four correct answers. From this result, the probability of detecting the star vibration at the fixation point was estimated as 99.1%, and this value was also >96.3%. Of the 120 trials in the gaze check tasks, there was no false-positive trial with “YES” response.

### Visual Field Test With Gaze Check Tasks

**Figure 3:**
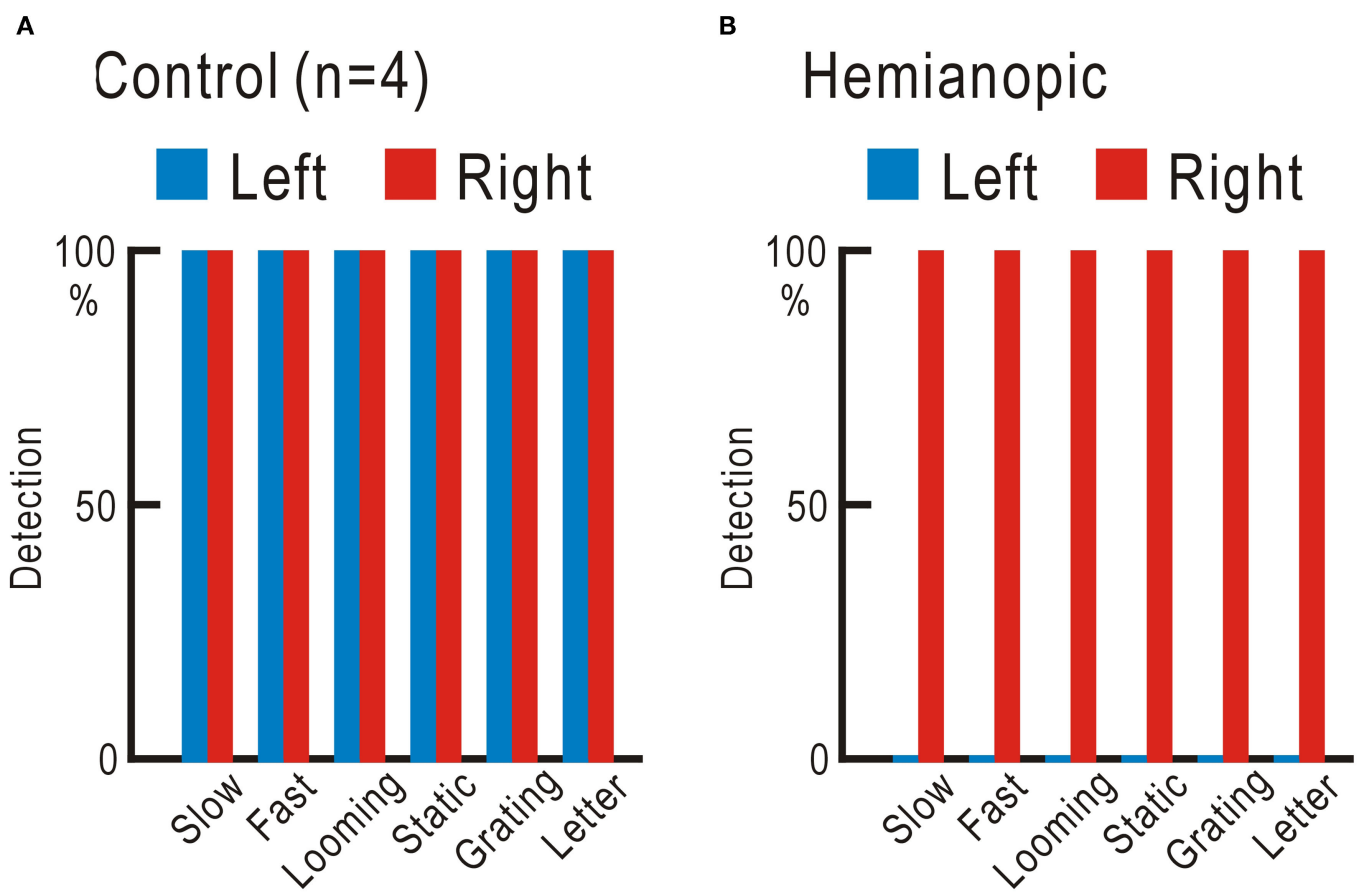
**(A)** Averaged results of four control participants. In both right and left visual fields, all six types of stimuli were fully detected 10 times in 10 trials. **(B)** Results of the homonymous hemianopic patient. All six types of stimuli were fully detected in the right visual field, but none was detected in the left visual field.

We tested the ability of the four control participants and one homonymous hemianopic patient using the visual field test with gaze check tasks. Six types of visual stimuli were applied: slow stimulus, a disk gradually appears and suddenly disappears; fast stimulus, a disk suddenly appears and gradually disappears; looming stimulus, a disk gradually enlarges; static stimulus, five disks appear; grating stimulus, vertical stripes appear and move to the right or left; and letter stimulus: one of the 46 Japanese Hiragana letters appears (Supplementary Video 1). The four control participants detected all six types of stimuli in both right and left visual fields 10 times in 10 trials for each stimulus (Figure 3A, Supplementary Table 2). The homonymous hemianopic patient detected all six stimuli in the right visual field, but no stimulus was detected in the left visual field (Figure 3B, Supplementary Table 2). The homonymous hemianopic patient made frequent mistakes when he tried to operate the computer-based visual field test by himself. Therefore, the results shown in Figure 3B were obtained by the patient orally answering “YES” or “NO,” while an examiner operated the computer to enter the responses. These results corroborated with those of Goldman perimetry (Figure 2B).

### Visual Field Test Without Gaze Check Tasks

**Figure 4:**
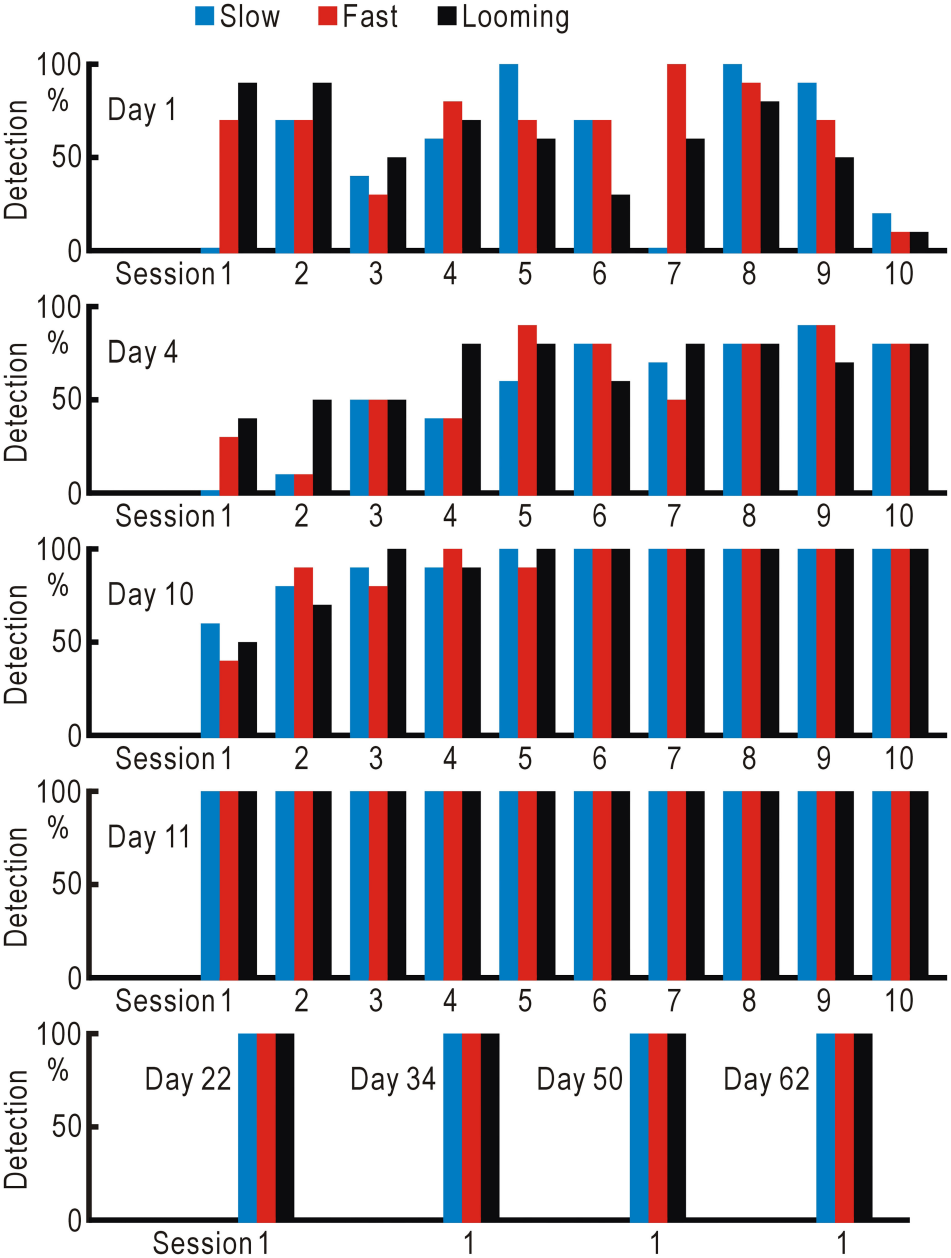
Training using the visual field test without gaze check tasks for the hemianopic patient. Slow (gradually appearing disc), fast (suddenly appearing and gradually disappearing disc), and looming (gradually enlarging disc) stimuli were repeatedly presented in 10 trials each, and the detection probabilities of the three types of stimuli were investigated.

We planned self-training using the visual field test to improve the visual defects, since repeated presentation of appropriate visual stimuli may ameliorate visual defects in hemianopic patients (12). However, if an extra examiner is required to operate the visual field test, self-training is not possible. Another concern was that the repeated mistakes by the patient might suggest the presence of some stress on the patient, and such stress could interfere with the training. Therefore, we omitted the gaze check tasks from the visual field test and found that he could operate the simplified test by himself with fewer mistakes. Using this test, the left visual field detectability of the slow, fast, and looming stimuli was tested in 10 trials each, and this sequence was repeated many times (Figure 4, Supplementary Table 3). On the first 4 days (days 1, 4, 10, and 11), 300 trials were tested on each day. The detection probability gradually increased, and the detection probability of all three types became 100% from the middle of the third day (day 10) and remained so thereafter.

**Figure 5:**
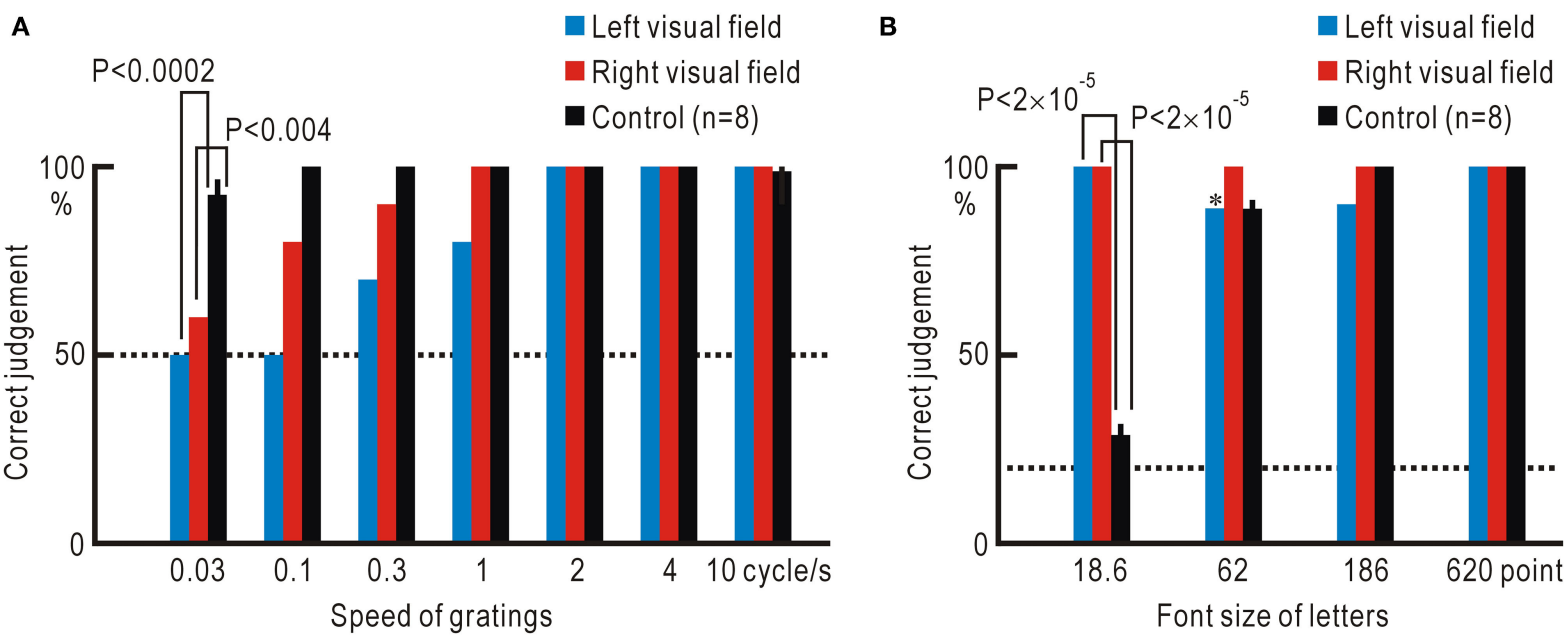
**(A)** Accuracy of the judgment regarding the direction of grating stimuli at various moving speeds. **(B)** Accuracy of reading letters in various font sizes. *Correct judgment was made in eight of the nine trials in which letter stimuli were detected. Dotted lines show the chance level of judgment.

The increased stimulus detection after repeated presentation of visual stimuli (Figure 4) suggested that some form of neural plasticity might be induced in the visual system (12). We further investigated the patient's visual functions in detail. We used moving grating patterns at a speed between 0.03 and 10 cycles/s (0.165 and 55.2°/s, respectively) and asked the patient to judge the movement direction (Figure 5A, Supplementary Table 4). The patient showed reduced ability to judge the direction of the slowly moving grating patterns in both the left and right visual fields. At the slowest speed of 0.03 cycles/s (0.165°/s), the control participants could judge the correct direction with a probability of 92.5 ± 4.5% (*n* = 8). However, in the hemianopic patient, this probability was reduced to almost chance level (left visual field: 50%, right visual field: 60%); these probabilities were significantly lower as compared to the control participants (left visual field: *P* <0.0002, right visual field: *P* <0.004).

Next, we investigated the letter-reading ability using Japanese Hiragana letters of a font size between 18.6 and 620 points (Figure 5B, Supplementary Table 5). The smallest letters of 18.6 point were hardly legible to the control participants, since the letters were presented ~22.25° from the fixation point; the probability of correct reading was 28.8 ± 3.0%, which was close to the chance level of 20%. In contrast, the probability of correct reading was significantly higher in the hemianopic patient with a 100% correct rate for both visual fields (left visual field: *P* <2 ×10^−5^, right visual field: *P* <2 ×10^−5^).

### Gaze Shifts During the Visual Field Test Without Gaze Check Tasks

These apparently bizarre results obtained using the visual field test without gaze check tasks are easily explained by the assumption that the patient could shift the gaze during the test. To judge the direction of slowly moving grating patterns, it is necessary to fix the gaze and sense the relative speed between the patterns and fixation point; however, this would be very difficult when the gaze is moving. Furthermore, small letters could be easily read if they were seen with the macular part of the visual field. Additionally, there would be no difference between the left and right results if the left and right stimuli were seen using the same part of the visual field on the healthy side.

**Figure 6:**
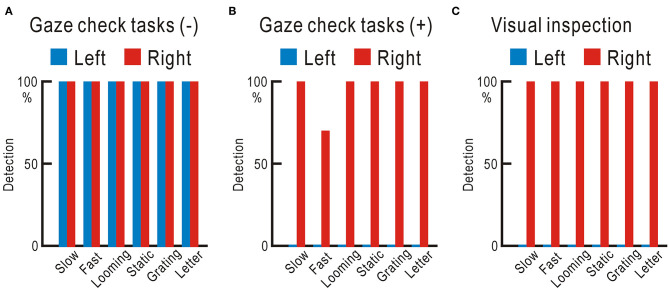
**(A)** Visual field test without gaze check tasks. Visual stimuli completely detected in both the left and right visual fields. **(B)** Visual field test with gaze check tasks. No visual stimulus detected in the left visual field. **(C)** Visual field test with visual inspection of the gaze. No visual stimulus detected in the left visual field.

The hemianopic patient detected all six types of visual stimuli without failure in either visual field, when investigated using the visual field test without gaze check tasks (Figure 6A, Supplementary Table 6). However, he could not detect any of the visual stimuli in the left visual field, when tested using the visual field test with gaze check tasks again (Figure 6B). For confirmation, the examiner directly checked the gaze during the test using visual inspection (12), and it was clear that the left visual field of the patient was not visible (Figure 6C).

The results so far strongly suggest that the gaze of the hemianopic patient was shifting during the visual field test without gaze check tasks. We tested this possibility by visualizing the eye movements. When visual stimuli were directed at the left visual field, leftward eye movements of the patient were observed as expected, while such eye movements were rarely found in a control participant (Supplementary Video 2). Remarkably, visual stimulation applied to the right visual field also induced similar eye movements in the patient (Supplementary Video 3). Few head movements were observed during the visual field test.

In order to investigate the detailed relationship between eye movements and visual stimuli, gaze shifts during the test were analyzed using an eye tracker (Tobii Pro Nano, Tobii Technology, Stockholm, Sweden), and the results were superimposed on the screen images of the visual field test. When visual stimuli were directed at the left visual field, the gaze moved in the upper left direction and scanned the stimulus presentation area (Supplementary Video 4). The timing of gaze shifts was not necessarily linked to the timing of the stimulus presentation. Even when the stimulus was directed at the right visual field, the gaze shifted toward the upper left direction (Supplementary Video 5), suggesting that the eye movements were involuntary.

**Figure 7:**
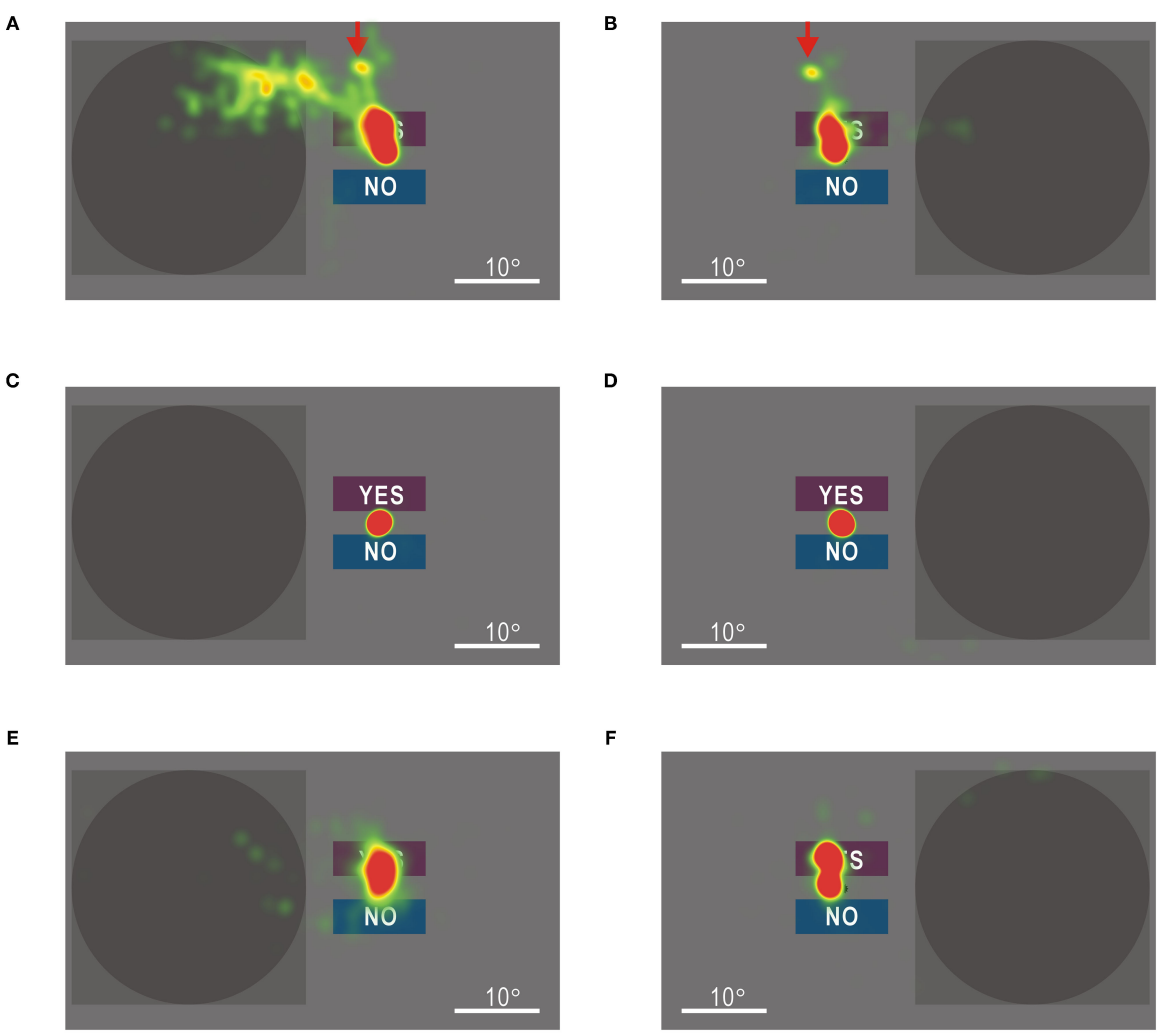
**(A)** Heat map of gaze shifts of the patient with homonymous hemianopia during the left visual field test without gaze check tasks. Color maps are shown in an arbitrary scale. **(B)** Gaze shifts of the patient during the right visual field test without gaze check tasks. The gaze shifts to the upper left direction in **(A)** were also observed (red arrows). **(C)** Gaze shifts of a control participant during the left visual field test without gaze check tasks. **(D)** Gaze shifts of the control participant during the right visual field test without gaze check tasks. **(E)** Gaze shifts of the patient during the left visual field test with gaze check tasks. **(F)** Gaze shifts of the patient during the right visual field test with gaze check tasks. Gaze shifts in **(E)** and **(F)** were more restricted around the fixation point than those in **(A)** and **(B)**, respectively.

The gaze shifts during the visual field tests are shown as heat maps. When gaze check tasks were omitted, the hemianopic patient showed gaze shifts in the upper left direction, regardless of stimulus presentation (red arrows in Figures 7A,B). When visual stimuli were presented in the impaired left visual field, additional gaze shifts were observed as if the stimulus presentation area was scanned (Figure 7A). In the control participant, the gaze remained around the fixation point (Figures 7C,D). Moreover, in the hemianopic patient, the gaze shifts were largely restricted around the fixation point in the visual field test with gaze check tasks (Figures 7E,F), indicating that gaze check tasks effectively suppressed involuntary gaze shifts. These results confirmed that the good performance of the hemianopic patient in the visual field test without gaze check tasks (Figure 6A) could be attributed mainly to gaze shifts.

## Discussion

There are several perimetry methods such as Goldmann perimeters, Humphrey perimeters, automated perimeters (25, 26), and computer-based perimeters (27). These methods allow us to investigate a map that displays which parts of the visual field are functional and which are impaired. However, they are not suitable for investigating the functional characteristics of a particular part of the visual field in response to various visual stimuli and changes in characteristics during rehabilitation of impaired visual functions. The visual field test in this study was developed for the latter purpose, and various visual stimuli can be applied to the left or right visual field (Supplementary Video 1). It is also possible to analyze the ability to judge the direction of moving grating patterns at various speeds and the ability to read letters of various font sizes. Generally, neurons in higher visual cortices have larger and more complex receptive fields as compared to those in the retina and primary visual cortex (13–15); therefore, the test in this study could be applied to evaluate the function of higher visual cortices. Some patients with cortical visual impairment can sense dynamic visual stimuli presented in the impaired visual field (28, 29), and training of these rudimentary visual functions may improve symptoms of cortical visual impairment (12). The visual field test of this study may be useful as a means of analyzing the properties of cortical visual impairment and self-rehabilitating the impaired visual functions.

In visual field tests, gaze control is required to apply visual stimuli to a specific part of the visual field. The image of the eyes has sufficient information for estimating the direction of the gaze, and this can be used for visual inspection of the gaze or eye tracking technology (12, 30). However, visual inspection of the gaze by an examiner is not suitable for self-rehabilitation of impaired visual functions. Estimation of the gaze using an eye tracker is influenced by the relationship between the head and screen, and it requires frequent calibration. Another way to confirm gaze fixation is to find the Marionette blind spot (Figure 2B); when the Marionette blind spot cannot be found, it is assumed that the gaze may not be fixed. However, this method can only be applied to test each eye separately, while it is preferable to evaluate binocular vision for estimating visual functions in daily life. In this study, we checked the position of the macula instead of the Marionette blind spot. The macula is only about 2° in diameter and has the best visual acuity (31). When we concentrate on detecting subtle vibrations of a small object, we try to see the image of the object using the macular part of the visual field. It is true that we can detect the subtle vibration in retinal areas other than the macula if we intentionally try to do so (Figure 1D). However, analysis of actual test data suggests that the four control participants and one hemianopic patient in this study detected the star vibration placed at the expected fixation point using the macula or the vicinity. Patients with homonymous hemianopia usually have preserved bilateral macular functions (24, 32). However, the macular area in the left visual field was impaired in the present patient, probably because of the large extent of the cortical lesion (Figure 2), so that the accuracy of gaze fixation might be reduced (33).

The present results suggest that the gaze during the visual field test was successfully limited within 4.7° from the fixation point. This estimation is based on a rough discussion that ignores individual differences and changes in participants' attention during the test. However, from the subjective experience of performing the visual field test with gaze check tasks, we believed that it was substantially impossible to have four or five correct answers in five gaze check trials without focusing gaze on the small star-shaped target at the fixation point. Furthermore, when compared with the heat maps of gaze shifts in the test without gaze check tasks (Figures 7A,B), that of the hemianopic patient showed a clearly limited distribution of gaze shifts during the test with gaze check tasks (Figures 7E,F). These results clearly show the effectiveness of the gaze check tasks for limiting the gaze around the fixation point.

The patient in this study was unaware of the visual field defects in daily life, regardless of the homonymous hemianopia established in the Goldmann perimetry. This discrepancy might be attributed to anosognosia, as observed in Anton syndrome (16–19). Anton syndrome occurs, usually when the cortical damage is bilateral, but it may occur after hemilateral damages (18), as in this case. The patient, however, could read the smallest letters both with the healthy right side and impaired left side of the visual field, when tested using the visual field test without gaze check tasks (Figure 5B), indicating that anosognosia of Anton syndrome cannot explain the unawareness of the visual field defects in the present case. Another possibility is that the patient might look at visual stimuli in response to the sudden appearance of the stimuli in the impaired left visual field. Dynamic visual stimuli are detectable even in patients with cortical visual impairment, known as Riddoch syndrome (28, 29). If gaze shifts toward visual stimuli were triggered in the patient in response to the sudden appearance of visual stimuli, it is no wonder that he could read the smallest letters (Figure 5B). However, it is unlikely that gaze shifts were triggered by the stimuli, since gaze shifts did not coincide with the timing of stimulus presentation (Supplementary Video 4). Furthermore, even when visual stimuli were directed at the right visual field, gaze shifts toward the upper left direction were observed (Figure 7B, Supplementary Video 5). These results strongly suggest that gaze shifts in the patient were involuntary and independent of stimulus presentation. Furthermore, when performing the visual field test with gaze check tasks, the patient made many operational errors. It may have been difficult for him to operate the computer accurately, probably because he must have had to focus on suppressing involuntary gaze shifts. The distribution of gaze shifts in the patient (Figures 7E,F) was wider than that in the control participant (Figures 7C,D). The reduced gaze accuracy in the patient could be attributed to insufficient suppression of involuntary gaze shifts, as well as to the macular impairment in the affected visual field (33).

Passive eye movements induce retinal image shifts. However, the present patient reported that he did not feel any shifts in the image of the outside world in daily life. Therefore, eye movements and visual information processing need to have an intimate link in the patient. It is known that such linkage is required to stabilize the visual images during eye movements in normal individuals (34–36). The patient reported that he felt a sense of discomfort in his vision after the onset of cerebral infarction, but gradually became less aware of it over a period of ~1 year. The patient also reported that he gradually experienced fewer episodes of collision with pedestrians with the impaired left side of vision while walking. It may take a year or more to master the intimate coordination between eye movements and visual information processing.

A characteristic feature of the hemianopic patient in this study was that he had satisfactory visual functions, except for the ability to judge the direction of slowly moving grating patterns (Figure 5). In other words, if a hemianopic patient learns involuntary gaze shifts covering the impaired visual field and an intimate coordination between the gaze shifts and visual information processing, the visual functions may be recovered up to a level sufficient for daily life. This patient might have learned such coordination probably because he actively performed numerous gaze shifts combined with visual information processing when reading books almost every day. As evidence for this speculation, gaze shifts in the upper left direction from the fixation point were frequently observed during the visual field test without gaze check tasks (red arrows in Figures 7A,B). Such gaze shifts could correspond to the eye movements required for the line feeds in Japanese books with vertical lines arranged from right to left. The advantage of this compensatory strategy for visual field defects is that it scans the entire visual field using intact parts of the retina, resulting in excellent visual acuity (Figure 5B). It has been reported that rehabilitation for homonymous hemianopia is effective when started within 6 months after cortical damage (3–6). However, since this patient suffered cerebral infarction 12 years back, training of gaze shifts coordinated with visual information processing may be useful even in cases where conventional rehabilitation for visual defects has failed.

## Data Availability Statement

The original contributions presented in the study are included in the article/Supplementary Material, further inquiries can be directed to the corresponding author/s.

## Ethics Statement

The studies involving human participants were reviewed and approved by Research Ethics Committee of Kashiwazaki General Hospital and Medical Center. The patients/participants provided their written informed consent to participate in this study.

## Author Contributions

KS designed the experiments and software, and mainly performed the experiments. TY, AH, and DT assisted with the experiments. KS, TY, AH, and DT also participated in the study as the control participants. SH and TN performed the data analyses and discussed the results. KS wrote the manuscript, and all authors discussed the results and edited the manuscript.

## Conflict of Interest

The authors declare that the research was conducted in the absence of any commercial or financial relationships that could be construed as a potential conflict of interest.
